# Case Report: Wide-to-Narrow QRS Tachycardia in a 3-Month-Old Infant

**DOI:** 10.3389/fcvm.2021.722376

**Published:** 2021-08-23

**Authors:** Liang Zhao, Song Yan, Tao Wang, Yimin Hua, Kaiyu Zhou

**Affiliations:** ^1^Department of Paediatric Cardiology, West China Second University Hospital, Sichuan University, Chengdu, China; ^2^The Cardiac Development and Early Intervention Unit, West China Institute of Women and Children's Health, West China Second University Hospital, Sichuan University, Chengdu, China; ^3^Key Laboratory of Birth Defects and Related Diseases of Women and Children (Sichuan University), Ministry of Education, Chengdu, China; ^4^Key Laboratory of Development and Diseases of Women and Children of Sichuan Province, West China Second University Hospital, Sichuan University, Chengdu, China

**Keywords:** transesophageal, electrophysiological, tachycardia, infant, Coumel law

## Abstract

**Introduction:** It is rare to find that wide QRS tachycardia automatically changes to narrow QRS tachycardia, and it is more difficult to clarify the mechanism.

**Case Report:** A 3-month-old infant with recurrent paroxysmal supraventricular tachycardia underwent transesophageal cardiac electrophysiological examination. The wide QRS tachycardia was induced by atrial RS_2_ stimulation, and it soon changed to narrow QRS tachycardia automatically. By the accurate measurement of esophageal lead, it was found that the electrocardiogram changes completely conform to Coumel law. The mechanism of wide and narrow QRS tachycardia was orthodromic atrioventricular reentrant tachycardia with or without ipsilateral functional bundle branch block, and the accessory pathway was defined as the left free wall-concealed accessory pathway.

**Conclusion:** Transesophageal cardiac electrophysiological examination can reveal some special electrophysiological phenomena, and its non-invasive nature is especially suitable for infants.

## Introduction

Clinically, it is rare to find that wide QRS tachycardia automatically changes to narrow QRS tachycardia, and it is more difficult to clarify the mechanism. This article reports a case of a 3-month-old infant who was found to have the above-mentioned ECG changes during the transesophageal cardiac electrophysiological examination (TECEE). Through accurate measurement of the esophageal leads, we confirmed that the changes in the electrocardiogram conform to Coumel law, an important electrophysiological phenomenon, and clarified the mechanism of tachycardia.

## Case Presentation

A 3-month-old infant, with a cough for 4 days and repeated paleness for 1 day, was admitted to the hospital. In the neonatal period, the infant was hospitalized in the neonatal department for sustained paroxysmal supraventricular tachycardia (SVT) and terminated with intravenous injection of adenosine triphosphate, and then followed by oral digoxin. After discharge, digoxin was discontinued because of the inadvisable opinion of the parent. The infant had a 6-year-old healthy brother and no history of tachycardia in his family. The mother of the infant was healthy during pregnancy, had no history of drug abuse, had no negative family background or events, and delivered at the age of 32. Admission examination of the infant revealed general conditions of wellness, a heart rate of 128 beats/min (bpm) with normal heart sound, regular cardiac rhythm and no heart murmur, as well as warm extremities,no swelling of the liver, and no edema in the lower limbs, and no abnormal results in the following routine: electrocardiogram, echocardiography, myocardial injury and serum electrolyte. After the family members signed the informed consent form, the child underwent a transesophageal cardiac electrophysiological examination. Due to recurrent tachycardia, the child underwent TECEE after the informed consent was signed by the parents.

After intravenous injection of Midazolam (0.2 mg/kg), the patient was sedated. The 7F esophageal electrode was inserted through the nose, and the DF-5A cardiac electrophysiological stimulation system was used for transesophageal atrial pacing.

**Figure 1:**
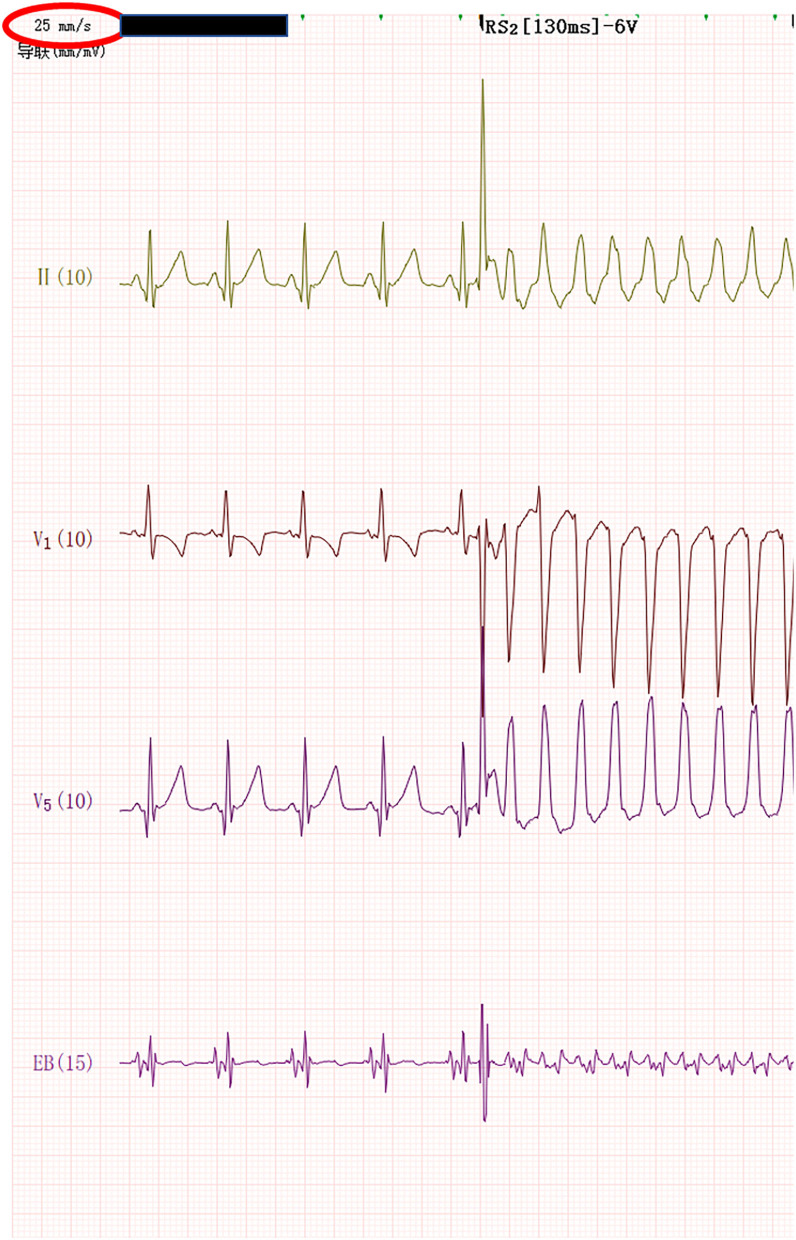
When atrial RS_2_ program stimulation reached 130 min; the wide QRS tachycardia of the left bundle branch block was induced at 259 bpm.

**Figure 2:**
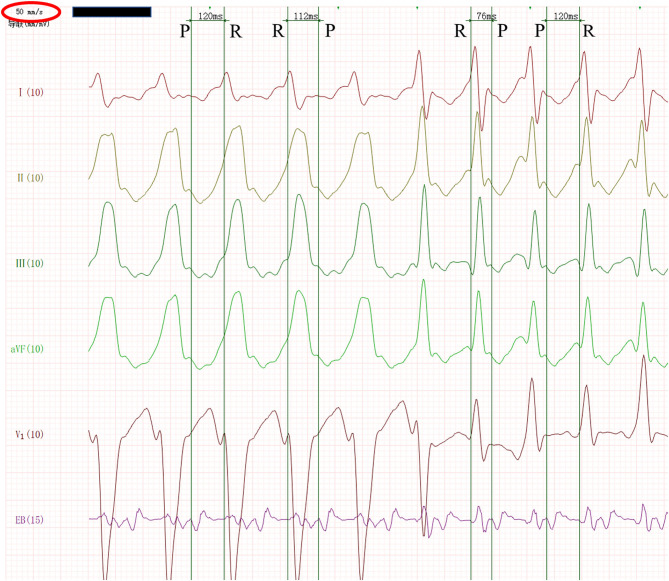
Wide QRS tachycardia suddenly and automatically changed to narrow QRS tachycardia. After zooming in the image and slowing down the paper speed to 50 mm/s, we measured the ECG in the esophageal lead. The RP interval was 112 ms, and the PR interval was 120 ms in wide QRS tachycardia, while the RP interval was 76 ms and the PR interval was 120 ms in narrow QRS tachycardia.

**Figure 3:**
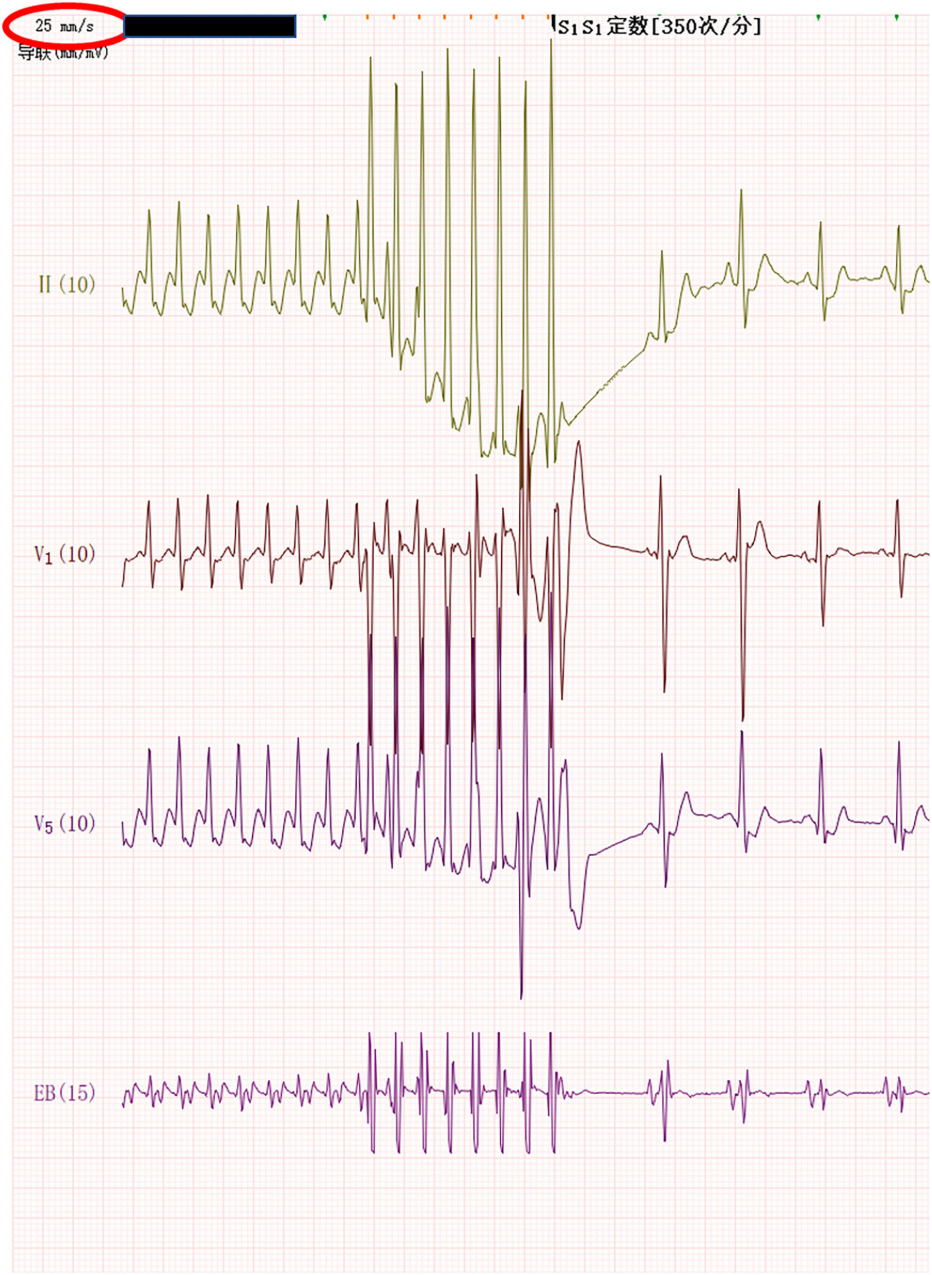
The tachycardia stopped abruptly after the atrium was captured by eight overdrive stimuli with a frequency of 350 bpm.

The infant was stimulated by atrial RS_2_ program. When the program stimulus negative scan to the combined rhythm interval was 130 ms, the S_2_-R interval was not significantly prolonged, and the wide QRS tachycardia of the left bundle branch block was induced at 259 bpm (Figure 1). The QRS wave time limit was 115 ms, the RP interval measured by the esophageal lead was fixed at 112 ms, and the PR interval was 120 ms. Sixteen seconds later, the tachycardia suddenly changed to a regular narrow QRS tachycardia of 306 bpm (Figure 2). The QRS wave time limit was 66 ms, and the P wave was inverted in lead I. The esophageal lead measured the RP interval of 76 ms and the PR interval of 120 ms. After capturing the atria with eight overspeed stimuli with a frequency of 350 bpm, the tachycardia suddenly stopped (Figure 3). There was no preexcitation pattern during sinus rhythm and atrial pacing.

During hospitalization, tachycardia did not occur again without special treatment. The infant was discharged after respiratory tract infection was cured. After discharge, the parents of the infant did not want him to take drugs for a long time to prevent tachycardia but required close follow-up. Once the tachycardia attack was found, it would be treated with drugs in time. After 3 months of follow-up, there was no recurrence of tachycardia.

## Discussion

Esophageal electrophysiological examination was adopted in this infant. Wide QRS tachycardia was unexpectedly induced during the programmed stimulation of atrial RS_2_, accompanied by restlessness in the infant. We needed to make a differential diagnosis immediately, especially to determine whether it was ventricular tachycardia or antidromic atrioventricular reentrant tachycardia, two wide QRS tachycardias that might quickly lead to hemodynamic instability. The ECG waveform of each lead was quickly observed. According to the four-step Brugada rule (1), the tachycardia did not have “absence of an RS complex in all precordial leads, no RS interval of the precordial lead more than 100 min” and there was no atrioventricular separation in the esophageal lead. According to the latest limb lead rule (LLA rule) (2), the tachycardia did not meet any of the following conditions: 1. monophasic R wave in lead aVR; 2. predominantly negative QRS in leads I, II, III; and 3. opposing QRS complex in the limb leads. In addition, the tachycardia was induced by atrial stimulation, and atrial stimulation rarely induced ventricular tachycardia. Therefore, the wide QRS complex tachycardia was insufficient evidence for ventricular tachycardia. Reviewing the induction process, there was no ECG waveform of ventricular preexcitation during the sinus rhythm and atrial RS_2_ program stimulation, so antidromic atrioventricular reentrant tachycardia could be basically ruled out.

Wide QRS complex tachycardia lasted 16 s, it was too short to take therapy, and the wide QRS tachycardia suddenly turned into a regular, typical narrow QRS SVT. We were relieved and then SVT was stopped by the conventional method of S_1_S_1_ overspeed stimulation.

We probed into this process of wide and narrow QRS tachycardia carefully, and measured the esophageal lead accurately, and found that the tachycardia was completely accorded with the typical Coumel law, which was an important electrocardiographic phenomenon.

The ECG manifestations of Coumel law mainly include the following: 1. There are two types of QRS complex in patients with orthodromic atrioventricular reentrant tachycardia: narrow QRS complex (excitation normal descending ventricle) and wide QRS complex (excitation descending with the left or right bundle branch block). 2. It was suggested that the ipsilateral block bundle branch of the accessory pathway, the prolongation of the RR interval, was mainly the prolongation of the RP interval, but the PR interval remained the same when the RR interval with the bundle branch block was longer than that of narrow QRS wave RR interval >35 ms. It was suggested that the accessory pathway was located on the opposite side of the bundle branch block if the RR interval of the bundle branch block was equal to the RR interval of the narrow QRS complex.

After zooming in the figure and slowing down the paper speed to 50 mm/s, we accurately measured the ECG in the esophageal lead. It was found that the RP interval was 112 ms and the PR interval was 120 ms in the wide QRS tachycardia. In the narrow QRS tachycardia, the RP interval was 76 ms and the PR interval was 120 ms. Both wide and narrow QRS tachycardia had the characteristics of RP interval less than the PR interval and RP interval was more than 70 ms, which was consistent with atrioventricular reentrant tachycardia. Further analysis showed that from wide QRS tachycardia to narrow QRS tachycardia, RP interval was shortened by 36 ms (more than 35 ms), while PR interval was completely unchanged. The change of QRS time limit was entirely due to the change of RP interval, which was in full compliance with Coumel law. Because Coumel law was only applicable to the bypass in the free wall of the left or right ventricle, and in the wide QRS tachycardia was a left bundle branch block pattern, the mechanism of wide and narrow QRS tachycardia was orthodromic atrioventricular reentrant tachycardia with or without the ipsilateral functional bundle branch block, and the accessory pathway was defined as the left free wall-concealed accessory pathway.

In this case, a non-invasive cardiac electrophysiological examination was performed for synchronous stimulation and recording. The process of inducing, changing, and terminating tachycardia and the characteristics of sudden abruptness were fully displayed, and typical figures conforming to Coumel law were collected. The RP and PR intervals of QRS complexes with different widths and narrowness were accurately measured so as to clarify the mechanism of tachycardia in the infant, locate the accessory pathway, and determine the direction for the future treatment of the infant.

Cardiac electrophysiological examination has important value for the determination of electrocardiographic phenomena (3, 4). Cardiac electrophysiological examination is divided into intracardiac electrophysiological examination and TECEE. The cases of wide QRS converted to narrow QRS tachycardia reported in the literature were mostly confirmed by intracardiac electrophysiological examination (5, 6). Compared with intracardiac electrophysiological examination, TECEE is simple, non-invasive, safe, inexpensive, and is especially suitable for infants who are temporarily unsuitable for radio frequency ablation.

TECEE can be carried out safely and effectively in infants and even newborns (7), which has important value in the diagnosis of supraventricular tachycardia and is worth promoting.

As far as we know, this is the first case report of automatic conversion of wide QRS to narrow QRS tachycardia in an infant, and the pathogenesis has been confirmed by the non-invasive examination method of TECEE.

## Conclusion

Transesophageal cardiac electrophysiological examination can actively induce some special electrophysiological phenomena, which can be reasonably explained through the analysis of esophageal lead. Its non-invasive nature makes it particularly suitable for infants.

## Data Availability Statement

The original contributions presented in the study are included in the article/supplementary materials, further inquiries can be directed to the corresponding author/s.

## Ethics Statement

The studies involving human participants were reviewed and approved by the Ethics Committee of West China Second University Hospital, Sichuan University. Written informed consent to participate in this study was provided by the participants' legal guardian/next of kin. Written informed consent was obtained from the minor(s)' legal guardian/next of kin for the publication of any potentially identifiable images or data included in this article.

## Author Contributions

KZ and LZ were responsible for the study design and manuscript preparation. SY, TW, and KZ were responsible for the clinical data. YH contributed to data acquisition. LZ wrote this manuscript. All authors contributed to the article and approved the submitted version.

## Conflict of Interest

The authors declare that the research was conducted in the absence of any commercial or financial relationships that could be construed as a potential conflict of interest.

## Publisher's Note

All claims expressed in this article are solely those of the authors and do not necessarily represent those of their affiliated organizations, or those of the publisher, the editors and the reviewers. Any product that may be evaluated in this article, or claim that may be made by its manufacturer, is not guaranteed or endorsed by the publisher.
